# Synthesis and applications of luminescent metal organic frameworks (MOFs) for sensing dipicolinic acid in biological and water samples: a review

**DOI:** 10.1039/d4na00652f

**Published:** 2024-11-21

**Authors:** Kawan F. Kayani, Omer B. A. Shatery, Sewara J. Mohammed, Harez Rashid Ahmed, Rebaz F. Hamarawf, Muhammad S. Mustafa

**Affiliations:** a Department of Chemistry, College of Science, Charmo University Peshawa Street, Chamchamal Sulaimani City 46023 Iraq; b Department of Chemistry, College of Science, University of Sulaimani Qliasan St 46002 Sulaimani City Kurdistan Region Iraq kawan.nasralddin@univsul.edu.iq; c Department of Anesthesia, College of Health Sciences, Cihan University Sulaimaniya Sulaymaniyah City Kurdistan Iraq; d Research and Development Center, University of Sulaimani Qlyasan Street, Kurdistan Regional Government Sulaymaniyah 46001 Iraq

## Abstract

The detection of trace quantities of 2,6-dipicolinic acid (DPA) in real-world samples is crucial for early disease diagnosis and routine health monitoring. Metal–organic frameworks (MOFs), recognized for their diverse structural architectures, have emerged as advanced multifunctional hybrid materials. One of the most notable properties of MOFs is their luminescence (L), which can arise from structural ligands, guest molecules, and emissive metal ions. Luminescent MOFs have shown significant promise as platforms for sensor design. This review highlights the application of luminescent MOFs in the detection of DPA in biological and aqueous environments. It provides a comprehensive discussion of the various detection strategies employed in luminescent MOF-based DPA sensors. Additionally, it explores the origins of L in MOFs, their synthesis, and the mechanisms underlying their sensing capabilities. The article also addresses key challenges and limitations in this field, offering practical insights for the development of efficient luminescent MOFs for DPA detection.

## Abstract 2

This review article discusses and summarizes the synthesis and applications of luminescent metal–organic frameworks (MOFs) for sensing dipicolinic acid in biological and water samples.

## Graphical abstract

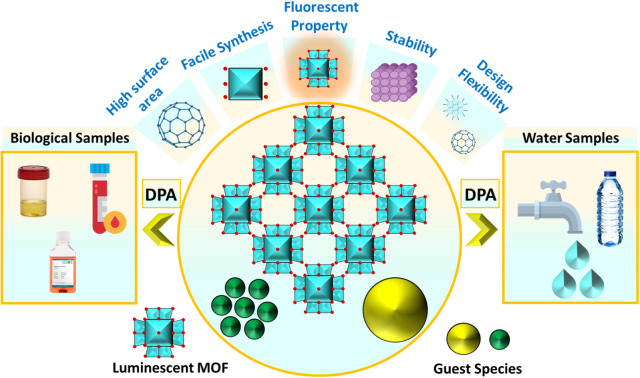

## 1. Introduction

Spores, a type of biological pollutant produced by bacteria, are highly dangerous to humans and hard to eliminate because they resist most common treatments.^1,2^*Bacillus anthracis*, a spore-forming bacterium, is the pathogen responsible for anthrax. This bacterium exists in two forms: rod-shaped organisms and spores. In nutrient-rich environments, the rod-shaped organisms grow and divide, but when nutrients are depleted, they convert into spores that can persist for decades.^3–6^*Bacillus* spores can contaminate food and water or be dispersed through aerosols, such as *via* air conditioning systems, posing a risk for both animal and human infections.^7^ Diagnosing anthrax is challenging because symptoms in humans can take 1–60 days to appear. *B. anthracis* spores are protected by several layers, with dipicolinic acid (DPA) being a key component, accounting for 5–15% of the spore's dry mass. Consequently, DPA serves as a unique biomarker for *B. anthracis*.^8–12^ However, detecting it is difficult because anthrax symptoms in humans may take up to 60 days to manifest.^13,14^

Currently, various approaches are used for detecting DPA, including high-performance liquid chromatography,^15^ surface-enhanced Raman spectroscopy,^12,16^ electrochemical methods,^17,18^ and fluorescence (FL).^19^ However, most existing DPA detection methods struggle to achieve both high sensitivity and a wide working range simultaneously.^20–22^ Therefore, it is essential to develop a detection system that is straightforward, fast, sensitive, and capable of detecting DPA across a broad range. Among these methods, FL detection stands out due to its affordability, sensitivity, speed, and the availability of portable instrumentation.^23,24^ Developing new fluorescent porous materials, particularly metal–organic frameworks, for DPA sensing is of great interest, as these materials offer stability and long emission lifetimes.^25^

MOFs, also referred to as porous coordination polymers (PCPs), are two- or three-dimensional porous crystalline materials (PCMs) characterized by infinite lattices.^26–30^ They consist of secondary building units (SBUs), metal cation salts or clusters, and polydentate organic ligands connected through coordination bonds.^31–33^ MOFs are a relatively new class of chemical materials with significant potential for sensor applications due to their large surface area, adjustable pore sizes, multiple functional sites, high stability, and ease of functionalization.^30,34–39^ Consequently, the tunable structural and surface properties of MOFs make them promising candidates for catalysis,^28,40^ sensing,^41–43^ drug delivery,^44,45^ gas separation,^46,47^ and the detection of toxic substances.^48,49^ Furthermore, MOFs have shown potential for onsite analysis and real sample analysis in various fields.^50^ Growing concerns about human safety have further motivated researchers to investigate MOFs for analytical applications.^51,52^

Luminescent MOFs represent a significant class of MOFs, regarded as promising candidates for sensor materials capable of detecting various substances, including heavy,^53,54^ molecules,^55,56^ and toxicants.^57^ These luminescent MOFs feature flexible structural units, with FL emanating from both the metal centers and ligands. Their optical properties can be modified through interactions among these components. In addition to the intrinsic fluorescence from MOF subunits, photo-responsive elements can be incorporated into MOFs to enhance FL.^58^ Recently, a wide array of luminescent MOFs has been synthesized using lanthanide elements,^59^ transition metals,^60^ and main group metal ions,^61^ along with luminescent sources such as carbon dots^62^ and dyes.^63^ However, advancing luminescent MOF-based sensors for practical applications in real-world scenarios remains an ongoing challenge. Further research is needed to develop new fluorescent MOF-based sensing materials for detecting DPA in human serum samples.

Several reviews have provided comprehensive overviews of MOF-based sensors, thoroughly summarizing the applications of luminescent MOF-based sensors.^64–68^ In this review, we focus on the various types of luminescent MOFs and their synthesis methods, and we provide a detailed summary of their sensing mechanisms. Additionally, we explore the latest advancements in the sensing capabilities of luminescent MOFs, particularly their effectiveness in detecting DPAs in biological specimens and water. Lastly, we critically examine the challenges and future prospects of MOF sensors, highlighting key points to demonstrate the growing interest in utilizing luminescent MOFs as sensors within the sensing field.

### 1.1 Scope of this study

Several reviews have been published on MOFs across various fields and quality assessments in the literature. For example, Khezerlou *et al.* summarized the use of MOFs for sensing tetracycline in food and water samples,^69^ while Raza *et al.* reviewed supercapacitor electrode materials based on BMOFs.^70^ Additionally, Luo *et al.* explored BMOFs for the detection of water contaminants.^71^

Despite the significance of previous studies, several key aspects, such as the design and strategies for utilizing BMOFs as sensors for DPA, have recently attracted increased attention. This review delves into the emerging field of BMOFs, offering a comprehensive analysis of their potential applications in DPA detection. We discuss DPA and its sources, the luminescence properties of BMOFs, various synthesis methods, and the sensing mechanisms. Additionally, the review explores the applications of BMOFs for DPA detection based on ratiometric systems, single-probe sensing, and visual detection methods. These insights contribute to a deeper understanding of how novel BMOF materials can function as DPA sensors, making this review both unique and relevant.

## 2. Dipicolinic acid and its samples

**Fig. 1:**
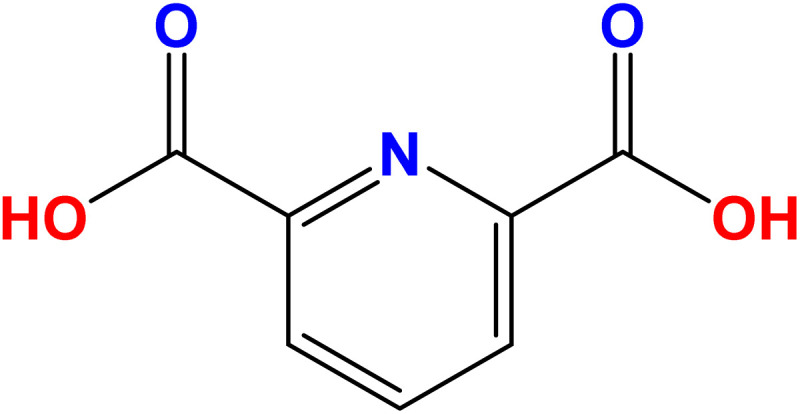
Chemical structure of DPA.

Dipicolinic acid (DPA), also known as pyridine-2,6-dicarboxylic acid (Fig. 1), is a key component of bacterial spores, comprising 5 to 15% of their total dry mass.^72^ Bacterial spores can release DPA during processes such as germination, hydrolysis, and heating. Consequently, DPA is commonly used as a biomarker for detecting the presence of *Bacillus* and *Clostridium* species in suspicious samples.^73^ Among the *Bacillus* species, *Bacillus anthracis* is the most toxic pathogen, with the 2001 anthrax attack in the United States resulting in five fatalities.^74^ Anthrax remains a significant public health concern, as the inhalation of approximately 10^4^*B. anthracis* spores can be fatal unless treated within 18 to 24 hours.^75^ While *Bacillus cereus* and *Bacillus subtilis* are less toxic than *B. anthracis*, they can still cause foodborne illnesses.^76^ Additionally, DPA itself has been associated with neurotoxic effects.^77^

Several techniques have been utilized for the analysis and detection of DPA, including high-performance liquid chromatography,^78^ capillary zone electrophoresis,^79^ and surface-enhanced Raman spectroscopy.^80^ While these methods offer high selectivity and sensitivity, they have certain drawbacks, such as being expensive, requiring extensive sample pretreatment, and involving complex procedures.^81^ Therefore, there is a need for a more cost-effective, user-friendly, time-efficient, and highly sensitive and selective method for detecting DPA in various matrices. Optical sensors have garnered significant interest due to their excellent selectivity, high sensitivity, simplicity, speed, and the ability to provide visible detection with the naked eye.^82–84^

In recent years, rapid advancements in nanotechnology have led to the development of innovative colorimetric and fluorescence sensors using various nanomaterials, including carbon dots,^85–87^ quantum dots,^88^ MOFs,^89,90^ and BMOFs.^91^ The scientific community has shown increasing interest in MOFs due to their porosity, structural diversity, adjustable compositions, and distinctive properties, which have made them widely applicable in sensing. Furthermore, BMOFs have gained increasing attention for their superior performance compared to single-metal MOFs and their mixtures in similar applications.^92^ As such, constructing a novel BMOF remains a formidable challenge for DPA sensing.

DPA is present in both biological and water samples. In biological samples, such as tissue or bodily fluids, DPA is released when bacterial spores germinate or break down. For instance, infections caused by *Bacillus anthracis* or *Clostridium difficile* can introduce spores into the body, where DPA may be detected during spore germination. Environmental exposure, such as through contaminated surfaces or air, can also result in spores entering biological systems.^93–95^ In water samples, DPA contamination typically occurs through runoff from soil, agricultural activities, or untreated wastewater, which may contain spores from spore-forming bacteria. Spores are highly resilient, allowing them to persist in water sources for extended periods, and their presence can indicate contamination from bacterial sources.^96^ DPA is used as a reliable biomarker to detect and quantify spore contamination in both biological and water environments.

As described in Table 1, this section summarizes traditional DPA detection methods and compares their performance with MOFs for DPA detection.

**Table 1:** Summary of traditional DPA detection methods and a comparison of their performance with MOFs for DPA detection

Detection method	Principle	Sensitivity	Selectivity	Advantages	Disadvantages	Comparison with MOFs
Fluorescence spectroscopy	Detection of DPA-induced fluorescence changes	Moderate to high	High	- Fast response	- Requires fluorophores	- MOFs can offer similar or higher sensitivity with tunable emission properties, and lower interference
				- High sensitivity	- Prone to interference	
UV-vis spectroscopy	Absorbance changes upon DPA binding	Moderate	Moderate	- Simple instrumentation	- Moderate selectivity	- MOFs offer improved selectivity through tailored pore structures and surface functionalization
				- Non-destructive	- May require large sample volumes	
Chromatography (HPLC)	Separation and quantification of DPA in mixtures	High	High	- Excellent selectivity	- Expensive equipment	- MOFs provide faster detection and do not require separation steps
				- Accurate quantification	- Time-consuming	
Mass spectrometry (MS)	Ionization and mass analysis of DPA	High	High	- High sensitivity	- Expensive equipment	- MOFs can achieve comparable sensitivity but with simpler, lower-cost detection mechanisms
				- Capable of detecting trace amounts	- Complex sample preparation	
Electrochemical methods	Current changes due to DPA redox activity	Moderate to high	Moderate to high	- Portable	- Requires conductive surfaces	- MOFs offer improved selectivity and avoid electrode fouling issues by using luminescence or capacitive sensing
				- Cost-effective	- May suffer from fouling	

## 3. Luminescent sensors

Luminescence-based sensing, which relies on changes in FL due to sensor–analyte interactions, has become a promising technique across various applications because of its high sensitivity, rapid response, and ease of use.^97,98^ Traditional luminescent sensors typically utilize organic dyes. However, the choice of sensor material is crucial for effective analyte detection. Conventional organic dyes used in luminescent sensors have several drawbacks, including toxicity, a tendency to aggregate, susceptibility to photobleaching, and limited adsorption capacity for target analytes.^64^ To address these issues, a range of L materials, such as metal complexes,^99^ carbon dots,^100,101^ nanoclusters,^102^ and lanthanide-doped inorganic phosphors,^103^ have been extensively explored.^104^ Recently, luminescent MOFs have garnered significant attention in both fundamental and practical research. These MOFs exhibit inherent luminescence, adjustable pore sizes, high adsorption capacities, and easily functionalizable surfaces, which enhance host–guest interactions and translate these interactions into detectable luminescence responses, making them an excellent material for fabricating fluorescent sensors.^105,106^ Typically, the luminescence performance of MOFs can be achieved through two main strategies. The first strategy involves synthesizing MOFs using luminescent metal ions (such as lanthanide ions like Eu^3+^, Ln^3+^, Tb^3+^, and Dy^3+^) as the coordination center, or using organic ligands that contain aromatic or conjugated moieties as linkers.^81^ The second strategy involves encapsulating luminescent guest molecules or luminescent nanoparticles within non-luminescent MOFs to trigger L.^107^

### 3.1 Origins of L in MOFs

**Fig. 2:**
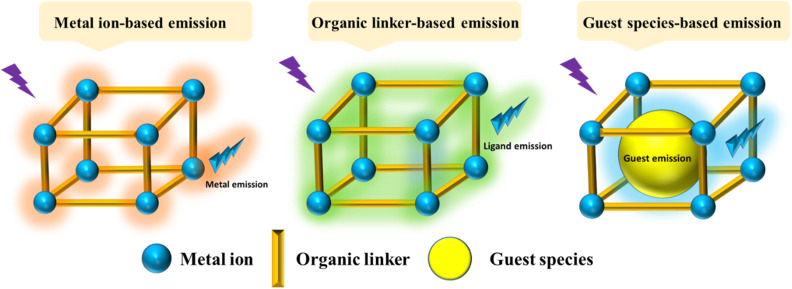
Illustration of potential emission modes in MOFs.

Recently, different structural architectures of MOFs have been created, showcasing their potential as multifunctional materials, particularly in designing luminescent sensors. The L of MOFs originates from organic ligands,^108^ guest species,^109^ and metal ions^110^ making it essential to choose suitable linkers, metal nodes, and guest molecules to design and synthesize MOFs with the best luminous properties (Fig. 2). Ligands provide antenna effects (AEs) for lanthanide MOFs (Ln-MOFs), and π-conjugated backbones are crucial for the L properties of MOFs.^111^ Although Ln ions and light-emitting organic linkers are commonly used to create luminescent MOFs, some non-luminescent MOFs have also shown significant potential as sensors. Another effective approach to develop unique fluorescent sensors using MOFs is to encapsulate luminous guest molecules within their pores.^112^

MOFs allow for the selection of metal ions and organic ligands with specific functionalities. FL can be introduced through metal ions, ligands, or guest molecules, with the FL enhanced by charge transfer (CT) between the ligands and metals.^113^ The L properties of these materials are influenced by various processes involving different ligands, including metal to ligand charge transfer (MLCT), ligand to metal charge transfer (LMCT), metal-based emission, ligand-based L emission, AEs, sensitivity, excimer or stimulated complex emission, adsorbate emission, and surface activity. The emission mechanisms of coordinated dual-emission MOFs primarily include metal-centered emission, and ligand-centered emission.^114,115^ The MLCT and LMCT processes depend on the relative energy levels of the lowest excited states in MOFs. If the energy level of the organic ligand's lowest excited state is lower than that of the metal ions, the CT from metal ions to organic ligands results in L, characterizing the MLCT process. Conversely, if the CT occurs from organic ligands to metal ions, it defines the LMCT process.^116^

#### 3.1.1 Metal ion-based emission

**Fig. 3:**
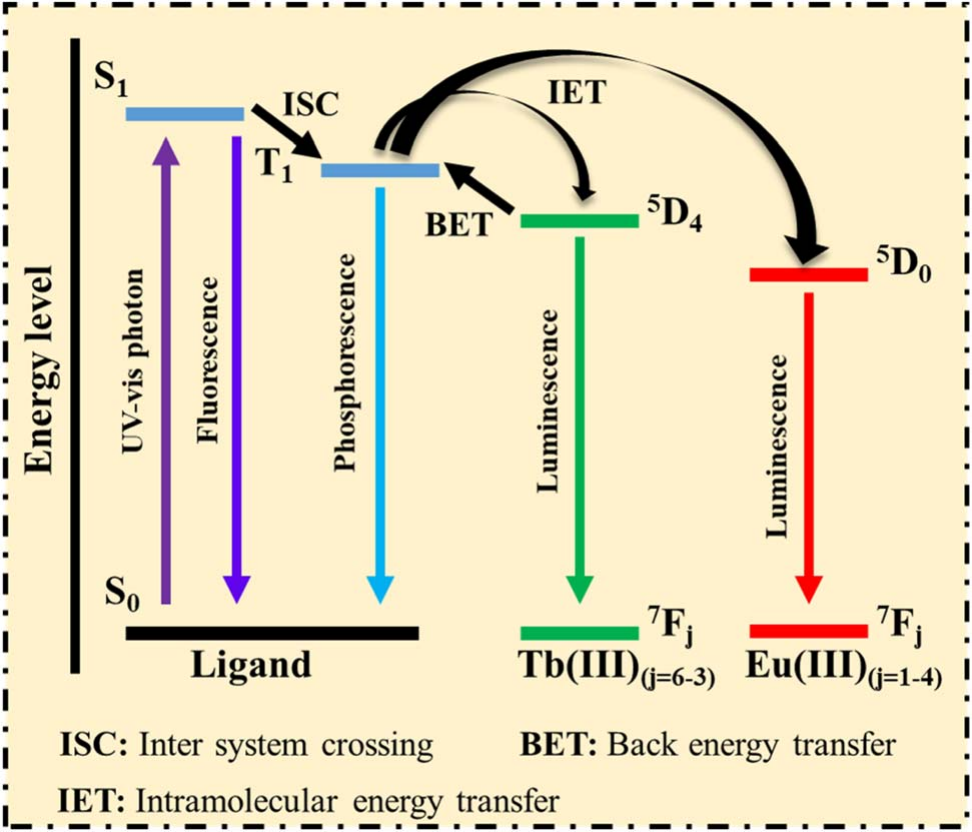
Energy absorption and transformation processes, adapted from ref. 121.

Extensive research has been conducted on Ln-MOFs as luminescent probes due to their long L lifetimes, high color purity, and significant Stokes shifts resulting from f–f transitions *via* an AE.^117^ In this process, organic ligands in Ln-MOFs function as ‘antennae’, absorbing photons from the UV-vis spectrum and transferring energy to the Ln ions.^118–120^ The mechanisms of energy absorption and transfer are illustrated in Fig. 3, with Eu^3+^ and Tb^3+^ chosen as representative Ln ions for a detailed explanation:

1. Ligands with aromatic or large conjugated systems absorb photons from the UV-visible spectrum, causing the ligand's ground state (S_0_) to transition to the singlet excited state (S_1_).

2. The unstable excited state of the ligands can release energy in two ways: first, the excited state S1 directly returns to the S_0_ state, emitting a photon. The second pathway involves the excited state S_1_ transferring energy to the triplet state (T_1_) *via* intersystem crossing (ISC), where the T_1_ state may return to the S_0_ state through phosphorescence (PH).

If the T_1_ state aligns with the energy level of Ln^3+^, the energy is transferred through intramolecular energy transfer (IET), effectively sensitizing Ln^3+^ emission. Subsequently, the excited Ln^3+^ returns to the S_0_ state, emitting its characteristic L. Thus, ligands with strong photon absorption are ideal for making Ln-MOFs, which serve as promising luminescent sensors.^121^

Yang *et al.*^122^ developed a novel probe for DPA detection based on a ratiometric system, where blue light-emitting Si nanoparticles (Si NPs) were encapsulated within green light-emitting Tb-MOFs. The Tb metal serves as the source of fluorescence signals.

#### 3.1.2 Linker-based (LB) emission

**Fig. 4:**
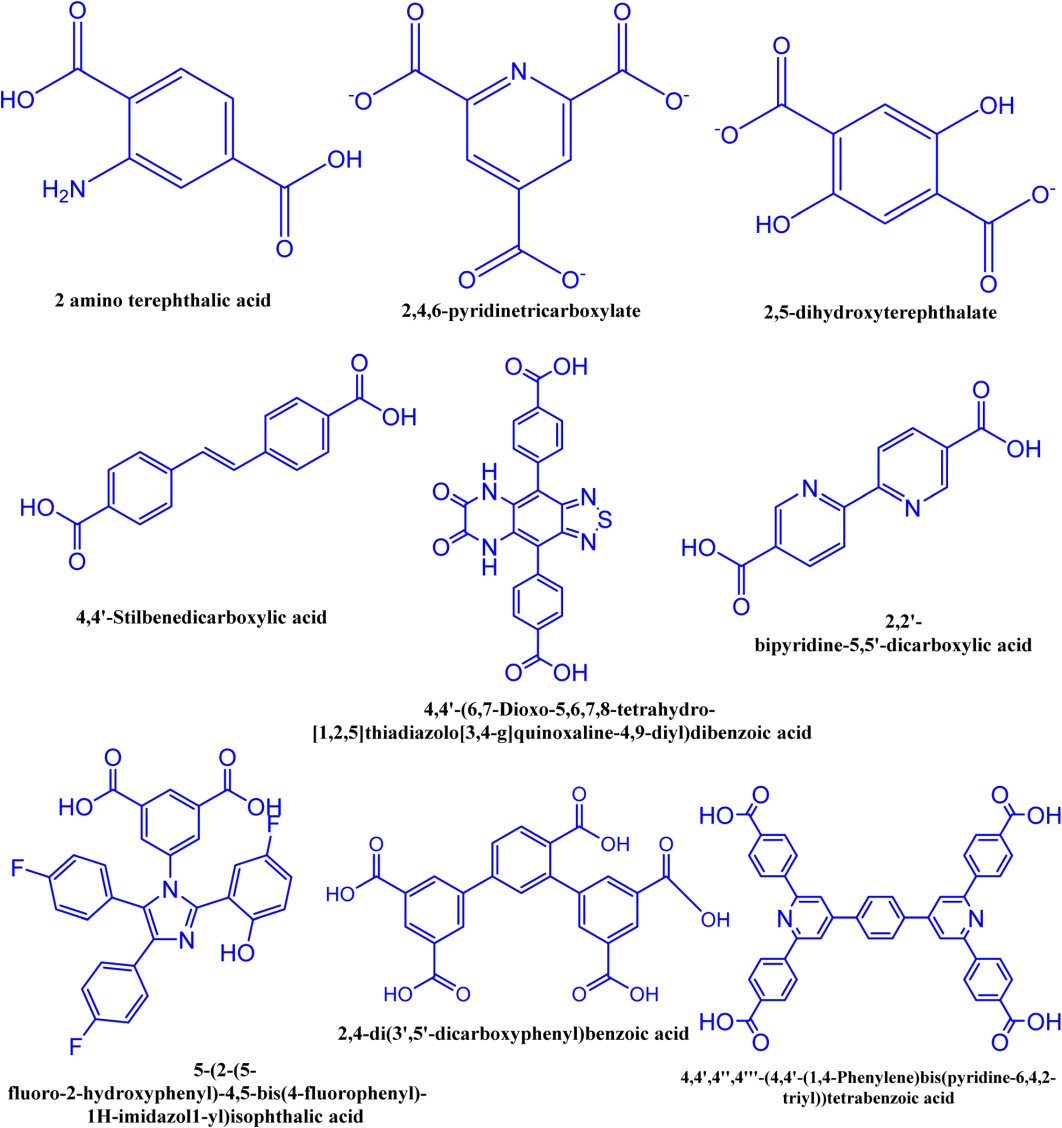
Some common organic linkers having luminescence characteristics.

LB emission in MOFs is achieved using various ligands and mainly relies on CT mechanisms, including MLCT, LLCT, and internal charge transfer of ligands (ILCT). MLCT L occurs when metal ions, irradiated by light, transfer energy to ligands, causing the S to shift from the metal to the ligand before returning to the S_0_ state and emitting FL. This process is common in complexes with oxidizable d^6^, d^8^, d^10^ electronic configurations, and p receptor ligands.^123^ The complex absorbs visible light, converting the excited MLCT state into a triplet MLCT state *via* intersystem crossing. FL emission occurs when electrons return from the excited MLCT state to S_0_, and PH emission occurs when they return from the triplet MLCT state to S_0_. The likelihood of MLCT increases with the reducibility of metal ions and the oxidizability of ligands. Organic ligands with π-conjugation significantly contribute to the L of LLCT. In this case, ligands form the framework's skeleton and are the main contributors to the characteristic emission of the MOFs.^124^Fig. 4 shows common organic linkers that exhibit luminescence.

Huo *et al.* developed a ratiometric probe using UiO-66-(COOH)_2_–NH_2_/Eu, which exhibits two emission peaks: one at 453 nm, originating from the 2-aminoterephthalic acid linker, which decreases progressively, and others at 598, 621, and 705 nm, which increase with the addition of DPA. As a result, it serves as a ratiometric fluorescence sensing platform for detecting DPA concentrations.^125^

#### 3.1.3 Guest molecule based emission

Quantum dots (QDs),^126^ dyes,^127–130^ and luminescent nanomaterials^23,131^ are some common luminescent guests that can enhance the FL properties of MOFs upon incorporation. These guest molecules, with customizable functional groups, provide effective interaction sites for binding with specific parts of analyte molecules. By leveraging the favorable design of guest-incorporated structures, researchers can develop more efficient guest@host systems.^132^

In addition, incorporating QDs, such as carbon dots (CDs),^84,133,134^ into a matrix creates a unique platform for CD-based sensors with significantly enhanced FL properties.^135^ Among various hosts for CDs, MOFs have shown favorable characteristics for loading guest molecules. Combining the inherent FL properties of CDs with the porous structure of MOFs allows for the detection of host–guest molecular interactions through changes in FL.^136^

Incorporating organic dyes into the structure of MOFs utilizes the nanospace within MOF pores as a molecular flask to create fluorescent host–guest materials. However, traditional MOF-based sensors, which rely on single-response FL, often suffer from inaccuracies and low sensitivity.^137^ To address these limitations, incorporating dyes into MOF pores (Dye@MOF) is a practical solution. Fluorescent dye molecules, which are cost-effective and easy to synthesize, are typically incorporated into luminescent MOF pores. Dye@MOF materials exhibit excellent FL behavior with dual emissive centers. For example, organic dyes such as fluorescein, and rhodamine, are known for their efficient fluorescence properties.^132,138^

Luminescent nanomaterials such as metal nanoclusters (NCs), have attracted significant interest due to their distinct physicochemical properties compared to conventional nanoparticles.^139^ NCs, which exhibit FL, consist of several atoms but behave molecularly due to their small size. These fluorescent NCs demonstrate surface plasmon resonance absorption in the visible light range and FL in the inner-infrared range. NCs are characterized by a long lifetime, large Stokes shift, good photostability, and excellent electrocatalysis properties.^140^ Additionally, incorporation of NCs into ZIF-8 pores enhances FL characteristics, resulting in improved FL lifetimes and higher detection efficiencies compared to traditional ZIF-8.^141,142^

Ma *et al.*^143^ designed a novel probe for detecting DPA. The initially weak red emission of Cu NCs is significantly enhanced by the addition of lanthanide Tb^3+^, attributed to the aggregation-induced emission (AIE) effect. This probe allows the monitoring of DPA due to the strong interaction between DPA and Tb^3+^, facilitated by the clamping configuration of the adjacent pyridine nitrogen and carboxylic acid groups; the addition of DPA causes the dissociation Tb^3+^ from the Cu NCs through a stronger coordination effect. This causes the Cu NCs to revert to a dispersed state, resulting in weakened fluorescence. Based on this mechanism, an “off-on-off” fluorescent probe for DPA detection was developed, where Tb^3+^ acts as a bridge to enhance the AIE fluorescence effect in Cu NCs and serves as a specific recognizer for DPA. This work highlights the potential of Cu NCs as a novel luminescent material.

## 4. Synthesis methods of MOFs

MOFs are composed of two main elements: metal ions and organic ligands or bridging linkers. MOFs are usually created by gently combining metal ions with organic linkers, producing materials that are both porous and crystalline.

### 4.1 Microwave assisted method

**Fig. 5:**
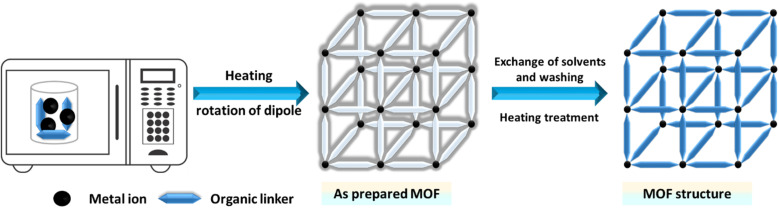
Microwave-assisted synthesis of MOFs.

Microwave assisted methods are extensively utilized for the rapid synthesis of MOFs under hydrothermal conditions, efficiently producing small metal and oxide particles.^144,145^ To achieve efficient heating, this method leverages the interaction between mobile solvent charges, such as polar solvent ions or molecules, and electromagnetic waves. Initially used in organic chemistry to prepare nanosized metal oxides, this technique involves filling a sealed Teflon vessel with a substrate mixture and an appropriate solvent, as illustrated in Fig. 5.^146,147^ The Teflon vessel is then microwaved for a specified duration and at a specific temperature. By aligning the permanent dipole moments of molecules with an electric field, the microwave quickly converts electromagnetic energy into thermal energy, rapidly heating the mixture.^148^ This energy-efficient heating technique increases the system's temperature and kinetic energy by generating molecular collisions.^149,150^ Achieving consistent nanocrystal sizes depends critically on the choice of solvent and energy input. Microwaves, ranging from 300 to 300 000 MHz, facilitate rapid crystallization and the formation of MOF products.^151–153^

### 4.2 Hydrothermal or solvothermal method

**Fig. 6:**
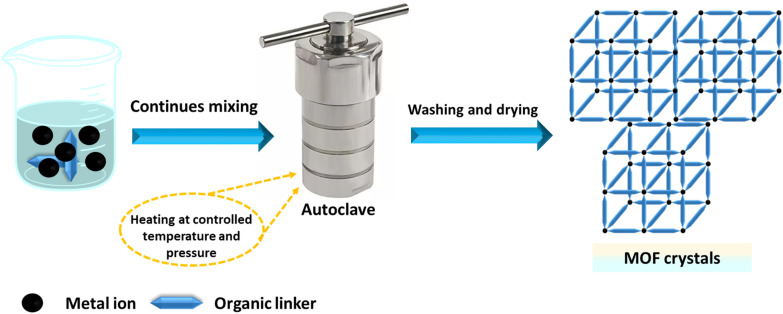
Schematic representation of the hydrothermal/solvothermal synthesis route.

The hydrothermal or solvothermal method is widely employed to prepare MOFs due to its simplicity, ease of use, and high crystallinity.^154^ This technique involves stirring metal salts and organic ligands in protic or aprotic organic solvents with formamide functionality. Aprotic solvents include dimethyl formamide, dimethyl sulfoxide, dimethylacetamide, among others,^155,156^ and protic solvents encompass methanol, ethanol, and various mixed solvents.^157^ To address solubility issues, solvent mixtures can be used. When water is used as the solvent, the process is called the hydrothermal method.^158^ The mixture is placed in a closed vessel at high pressure and temperature for several hours or a day, using glass vials for low temperatures and autoclave reactors for high temperatures. The closed vessel is heated above the solvent's boiling point to increase pressure.^159^ The key parameter is temperature: it must be above the boiling point under self-generated pressure for solvothermal reactions and below or at the boiling point under ambient pressure for non-solvothermal reactions. Under high pressure, the solvent can reach temperatures above its boiling point, which melts the salt and promotes the reaction. To achieve large crystals with a high internal surface area, slow crystallization from the solution is essential,^160^ as depicted in Fig. 6.

### 4.3 Mechanochemical methods

**Fig. 7:**
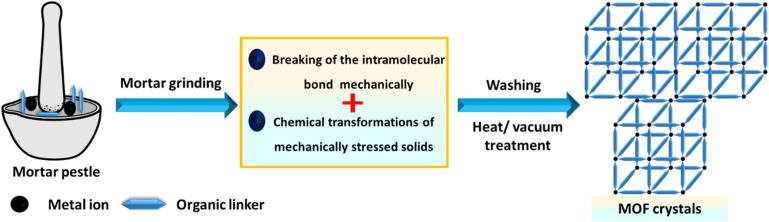
Mechanochemical synthesis of MOF structures.

Mechanochemistry involves using a ball mill or a mortar and pestle to introduce mechanical energy. Although this approach has been infrequently applied in MOF synthesis, it offers advantages such as simplicity, minimal or no solvent use, and reduced waste production.^161,162^ Certain MOFs can be rapidly synthesized by mechanochemically reacting the appropriate metal salt with an organic ligand, often with little to no solvent,^163,164^ as shown in Fig. 7. However, soluble metal salts are typically required for these mechanochemical processes.^165^

### 4.4 Sonochemical methods

**Fig. 8:**
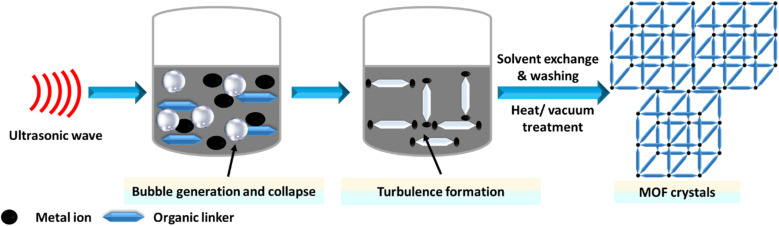
Sonochemical synthesis of MOF structures.

Sonochemistry studies the chemical reactions that occur when a reaction mixture is exposed to high-energy ultrasound. The primary aim of sonochemical synthesis in MOF research is to develop a rapid, eco-friendly, and room-temperature method that is easy to perform.^89,166^ This is particularly significant for future applications, as quick reactions could facilitate the scale-up of MOFs.^167,168^ Additionally, the nanocrystalline particles often produced by sonochemical methods are expected to be beneficial for their use. Systematic investigations have focused on the influence of reaction time, temperature, and solvent.^169,170^ It was found that short reaction times at ambient pressure resulted in high yields of the product.^171^ For example, Da-Won Jung successfully synthesized high-quality MOF-177 crystals ranging from 5 to 20 μm using a sonochemical method, significantly reducing the synthesis time. This process utilized the low-cost solvent 1-methyl-2-pyrrolidone, which has the highest CO_2_ adsorption capacity.^172^ The sonochemical synthesis of MOFs is illustrated in Fig. 8.

### 4.5 Electrochemical methods

**Fig. 9:**
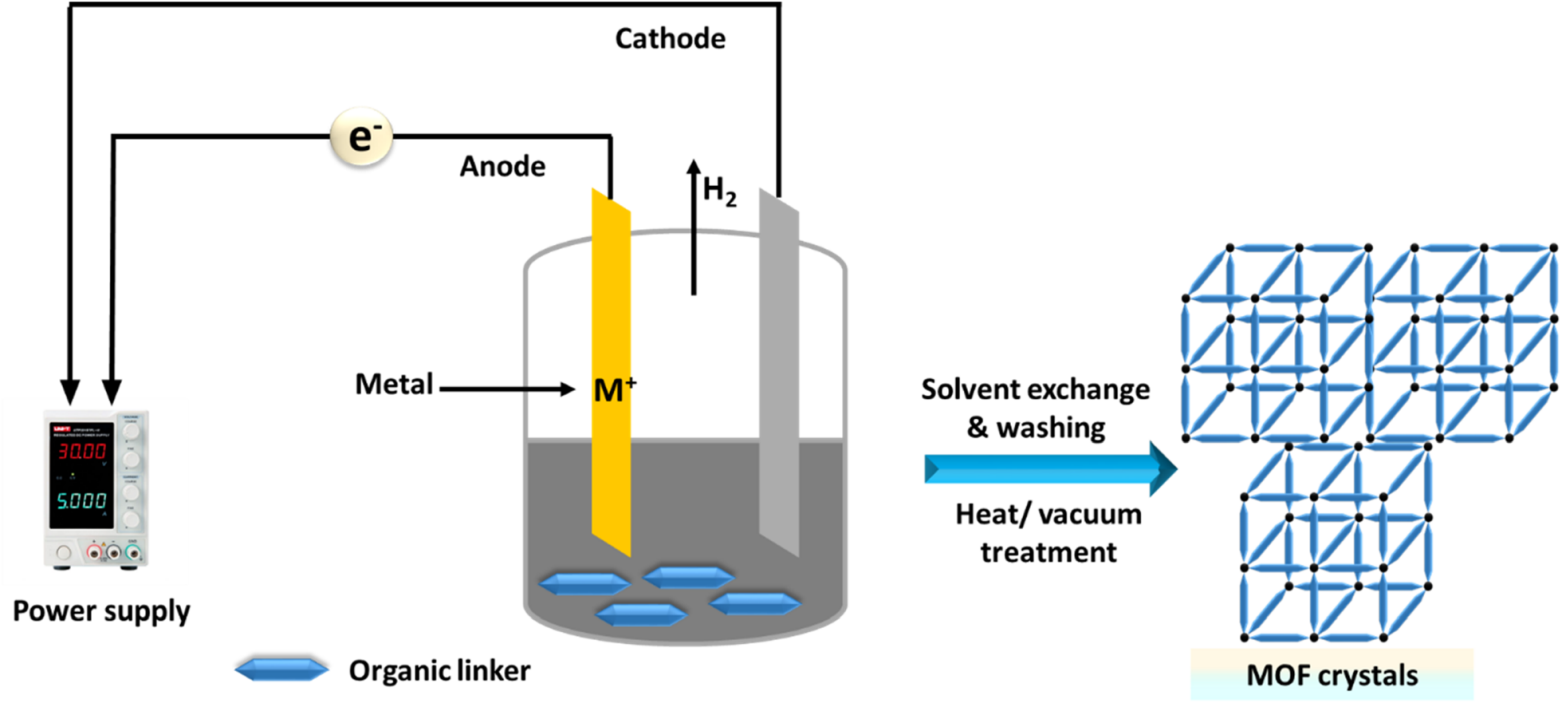
Electrochemical synthesis of MOFs.

Finally, we highlight the advancements in the electrochemical synthesis of MOF materials, detailing both anodic and cathodic methods.^173,174^ Electrodeposition is a simple, cost-effective technique that requires milder reaction conditions and shorter times.^175^ Rapid CT during synthesis results in the quick nucleation and growth of MOF crystals on substrates.^176^ This method enables the production of MOF thin films with adjustable morphology and crystallite size on various conductive substrates.^177,178^ The crystallinity and orientation of the thin films, which are crucial for practical applications, can be controlled by adjusting factors such as the applied potential energy, temperature, electrolyte, and solvent.^179^ The electrochemical synthesis of MOFs is illustrated in Fig. 9.

Alternative methods, including layer by layer assembly,^180^ spray-drying,^181^ diffusion,^182^ template strategies,^183^ post-synthetic modification^184^ and microemulsion,^185^ can be employed to synthesize various types of MOFs.

## 5. The sensing mechanisms of luminescent MOFs

In this section, we explain the sensing mechanisms of CM, PET, FRET, CA, and IFE, along with their advantages, disadvantages, and relationships with other mechanisms, as described in Table 2.

**Table 2:** A comprehensive comparison of CM, PET, FRET, CA, and IFE in terms of their full forms, advantages, disadvantages, and relationships with other mechanisms

Mechanisms	Full form	Advantages	Disadvantages	Relationships with other mechanisms
CM	Collapse mechanism	- Sensitive to environmental changes (*e.g.*, pressure, temperature)	- May be irreversible	- Can trigger or result from other mechanisms like PET and FRET if structural collapse alters energy or electron transfer pathways
		- Direct correlation with structural integrity	- Limited to MOFs with specific structural flexibility	
PET	Photoelectron transfer	- Highly selective	- Requires specific electron donor–acceptor pairs	- PET can compete with FRET and IFE mechanisms, as they all rely on changes in energy transfer or quenching
		- Strong quenching effect due to electron transfer	- Sensitivity limited to molecules that can engage in electron transfer	
FRET	Förster resonance energy transfer	- High sensitivity	- Requires spectral overlap between the donor and acceptor	- Can be influenced by structural collapse, which might change donor–acceptor proximity
		- Capable of detecting interactions over long distances (up to 10 nm)	- Distance-dependent efficiency	- Competes with PET when both energy and electron transfer occur
CA	Competition absorption	- Simple to implement	- Requires analyte with overlapping absorption spectra	- Similar to IFE, but primarily involves the analyte's absorption of excitation energy, whereas IFE involves reabsorption of emitted light
		- Efficient in quenching luminescence through absorption of excitation/emission by the analyte	- Limited selectivity	
IFE	Inner filter effect	- Does not require direct interaction with the MOF	- Can be confused with true quenching	- Often confused with CA but distinguished by its focus on reabsorption of emitted light
		- Effective for strongly absorbing species	- Dependent on analyte concentration and optical density	- Can occur alongside PET or FRET mechanisms, altering luminescence signals

### 5.1 Collapse mechanism (structural transformation, ST)

**Fig. 10:**
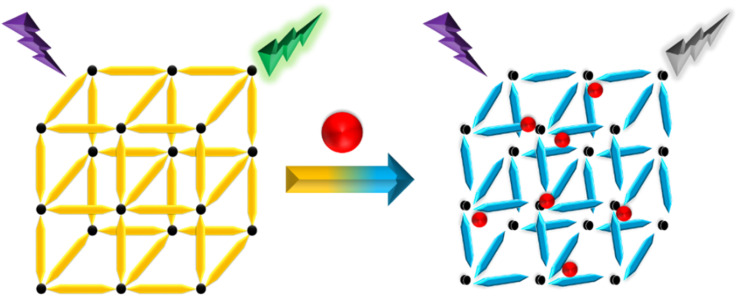
Schematic diagram of the ST mechanism.

The collapse of frameworks refers to the breakdown and transformation of the crystalline structure of luminescent MOFs into free ligands and metal ions following the detection of analytes.^186,187^ This process can be readily identified using powder X-ray diffraction (PXRD), and ICP analyses. After luminescence detection, the X-ray diffraction (XRD) pattern may show new peaks or the disappearance of existing ones, indicating the formation of a new structure.^188,189^ This structural change may lead to the loss of metal–ligand charge transfer emission or the emergence of ligand-based emission, resulting in significant photoluminescence (PL) changes in MOFs. This framework collapse mechanism poses challenges for reversibility experiments, rendering the MOFs non-reusable (Fig. 10).^190,191^ Pengyan Wu *et al.* synthesized a luminescent MOF using a hydrothermal method for the quantification of Hg^2+^. The presence of Hg^2+^ completely quenched the L of the luminescent MOF. Time-dependent PXRD patterns indicated that the crystalline structure of the luminescent MOF collapsed and transformed into a free ligand in the Hg^2+^ aqueous solution. This transformation was further supported by IR spectra, EA, and ICP.^186^

### 5.2 Photoelectron transfer (PET) mechanism

**Fig. 11:**
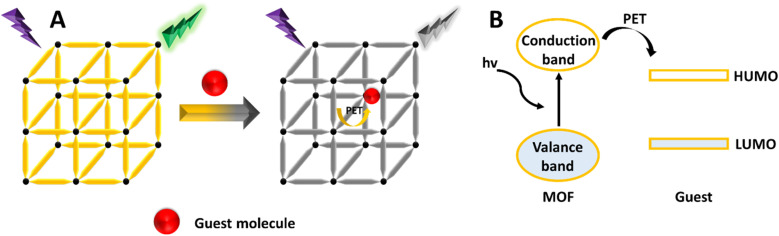
(A) Schematic illustration of the PET mechanism. (B) Diagram showing molecular orbitals.

PET is a process involving charge transfer in an excited state, where a photoelectron moves from an excited donor to a ground-state acceptor.^192,193^ If the donor's lowest unoccupied molecular orbital (LUMO) has higher energy compared to the acceptor's LUMO, the photoelectron will transfer to the ground-state acceptor instead of returning to the donor's ground state. This results in the quenching of the donor's emission.^194^ This PL sensing mechanism has been used to detect various analytes^43,195^ and pesticides.^196,197^ As shown in Fig. 11, when the energy level of the MOF's conduction band or LUMO exceeds that of the analyte's LUMO, photoelectrons can efficiently move from the MOF to the analyte. This process can quench the L of the MOF, serving as a signal for the presence of the analyte. Pan *et al.* developed a new Zn-MOF designed as a fluorescent sensor for detecting nitrobenzene using the PET mechanism.^198^

### 5.3 FRET (Förster resonance energy transfer) mechanism

**Fig. 12:**
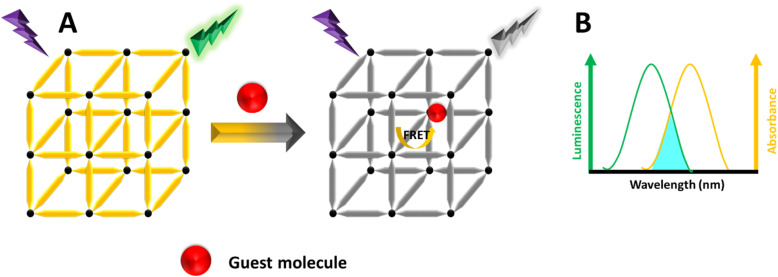
(A) Schematic illustration of the FRET mechanism. (B) The MOF's emission spectrum and the guest's UV-vis absorption spectrum.

FRET is a distance-dependent non-radiative energy transfer process, widely used in FL sensing.^199,200^ This phenomenon occurs when the emission spectrum of a donor molecule partially overlaps with the absorption spectrum of an acceptor molecule, enabling energy transfer from the donor to the acceptor (Fig. 12).^201,202^ The effectiveness of FRET is affected by factors such as the extent of spectral overlap, the distance between the donor and acceptor, and dipole–dipole interactions. When the excitation spectrum of target molecules overlaps with the emission spectrum of MOFs, the presence of these target molecules (acceptors) can alter the FL of the MOFs (donors).^124^ There are two main types of non-radiative energy transfer: Förster and Dexter mechanisms. FRET is a short-range mechanism that requires significant spectral overlap between the donor's emission and the acceptor's absorption spectra for efficient transfer.^203^ In contrast, Dexter energy transfer depends on the orbital overlap between the donor and acceptor, involving an electron exchange process, and its rate decreases exponentially with distance.^204^ Understanding these mechanisms is essential for designing energy transfer systems in LMOFs, facilitating the development of singlet–singlet or triplet–triplet energy transfer between linkers, metal centers, and guest molecules.^205^

### 5.4 Competition absorption (CA) mechanism

**Fig. 13:**
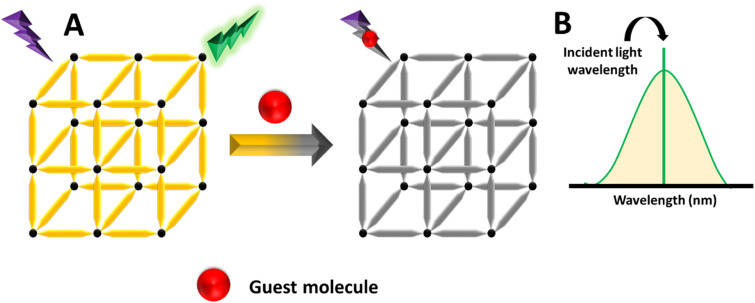
(A) Diagram illustrating the CA mechanism. (B) UV-visible absorption spectrum of the guest species.

When the absorption spectrum of an analyte coincides with the excitation spectrum of a MOF, both the MOF and the analyte compete for the excitation light.^206^ This competition causes the analyte to absorb some of the excitation light, thereby reducing the total energy available to the MOF.^207^ As a consequence, fewer excited states are populated in the MOF, leading to luminescence quenching of the MOF, as illustrated in Fig. 13. This mechanism is frequently proposed for the detection of Fe^3+^ ions^208^ and certain volatile organic compounds (VOCs) such as acetone,^209^ and the detection of 6-mercaptopurine.^210^

### 5.5 The inner filter effect (IFE) mechanism

**Fig. 14:**
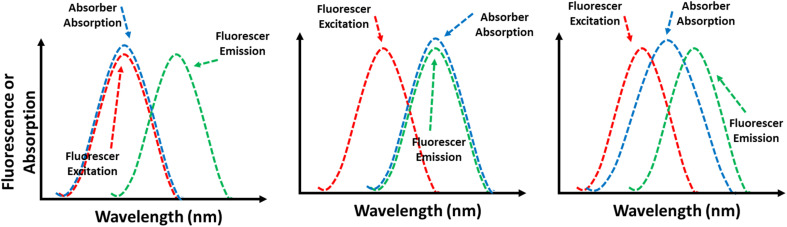
Schematic diagram of IFEs.

IFE happens when the absorption spectrum of a quencher in the detection system overlaps with either the excitation or emission spectra of LMOFs.^211,212^ Sometimes called apparent quenching, IFE is not a genuine quenching process. Instead, it arises from the attenuation of the excitation beam or the absorption of emitted radiation due to a high concentration of either luminescent MOFs or the quencher in solution.^213^ This effect results in a decrease in the intensity of the fluorescent MOF.^214^ The FL response to the analyte is significantly more sensitive than the UV-vis absorption at low concentration levels, resulting in a substantial improvement in detection sensitivity.^215^ In this mechanism, the absorption spectrum of the absorber may overlap with the excitation spectrum, emission spectrum, or both the excitation and emission spectra of the fluorescer, as shown in Fig. 14.

## 6. Applications

Measuring various analytes in serum such as metal ions,^216,217^ biomolecules,^218,219^ vitamins,^220,221^ and hazardous compounds,^222–224^ as well as in urine and other clinical samples, is essential for disease detection, monitoring, and management.^225^ Additionally, there is a significant demand to understand environmental conditions, particularly water quality, to detect hazardous substances in water samples.^226^ Fluorescence analysis using fluorescent sensors is effective for both qualitative and quantitative analysis of biological and chemical substances.^227^

FL-based sensors offer advantages over other analytical techniques such as electrochemical method,^228^ high-performance liquid chromatography,^229^ and atomic absorption spectroscopy,^230^ and mass spectroscopy,^231,232^ due to their cost-effectiveness, ease of operation, and high sensitivity.^233–237^ These sensors often utilize organic molecules,^238^ dyes,^239^ and fluorescent nanomaterials.^240–242^ Among the most commonly used fluorescent sensors are MOFs.^243–245^ Fluorescent MOFs, or luminescent MOFs, have garnered significant attention due to their unique properties, including controllable surface and pore sizes and excellent optical characteristics.^246,247^ As a result, a wide range of luminescent MOF-based sensors with diverse detection capabilities can be readily designed and implemented.^136–141^ Fluorescent DPA sensors based on luminescent MOFs have found widespread application in biomedical analysis and environmental water samples, as described below.

### 6.1 Ratiometric sensing

Over the past decade, fluorescence-based sensors have gained increasing attention for monitoring applications due to their high sensitivity, ease of use, and quick response time.^248–250^ However, quantifying a target analyte with fluorescent probes that exhibit single emission features presents significant challenges. Various analyte-independent factors such as instrumental parameters, the microenvironments around the probes, local concentrations of probe molecules, and photobleaching complicate precise analysis. The most effective way to address these challenges and ensure reliability is through ratiometric approaches.^83^ However, during FL intensity measurement, factors like concentration, environmental conditions, and excitation light intensity can reduce the accuracy of MOF-based monochromatic FL sensors.^251^ To mitigate this issue, an additional FL signal is introduced to create a MOF-based ratiometric FL sensor. The emission intensities at two wavelengths are independent of these interfering factors, enabling RF sensors to overcome the limitations of single FL sensing by self-calibrating dual-emission, thereby achieving accurate detection.^116^ Developing a new ratiometric sensing method is highly promising and critically important for the convenient detection of DPA in serum and water samples.

**Fig. 15:**
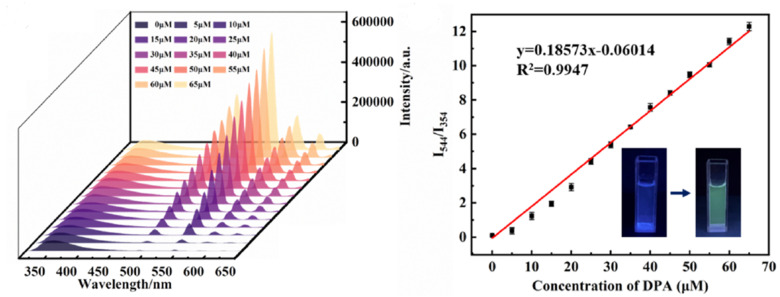
Schematic representation of the preparation of Tb^3+^@Ag-tpt and its application. Adapted from ref. 252 with permission. Copyright 2023, Elsevier.

In 2023, Yin *et al.* developed and synthesized two silver-based MOFs for monitoring DPA. The ratiometric FL probe Tb^3+^@Ag-tpt was created to detect DPA in aqueous solutions. Under 315 nm excitation, the FL spectrum of Tb^3+^@Ag-tpt revealed two L centers corresponding to the emissions of Tb^3+^ ions and Ag-tpt. Additionally, narrower, weaker peaks at 488, 544, 582, and 620 nm were linked to the transitions of Tb^3+^ ions, while an emission peak at 354 nm was associated with Ag-tpt. This probe demonstrated a working range of 0–65 μM and a low detection limit (LOD = 24.2 nM). Moreover, Tb^3+^@Ag-tpt showed a low LOD for bacterial spores (1.9 × 10^−4^ spores per mL). Consequently, these silver-based composite materials offer promising potential for reducing bacterial contamination and real-time detection of bacterial spores,^252^ as shown in Fig. 15.

Huo *et al.* created a ratiometric probe using UiO-66-(COOH)_2_–NH_2_/Eu, which displays two emission peaks: one at 453 nm that gradually decreases and others at 598, 621, and 705 nm that increase with the addition of DPA. Consequently, it functions as a ratiometric FL sensing platform for detecting DPA concentration. This platform demonstrated a reliable linear response (0.2–40 μM), with a detection limit of 25.0 nM, and exhibited a significant FL color change from blue to red, showing great potential for practical applications in river water and human serum analysis.^125^

**Fig. 16:**
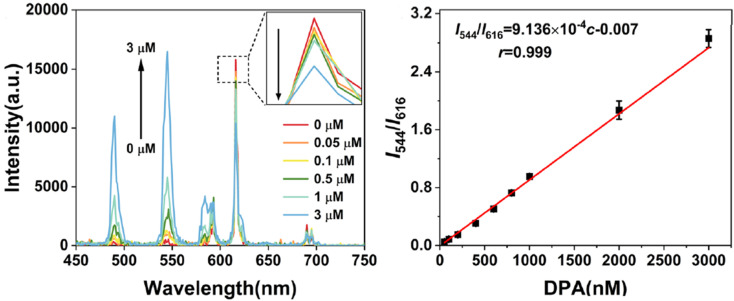
Schematic of the Tb/Eu-BTC sensor for DPA detection. Adapted from ref. 253 with permission. Copyright 2023, Elsevier.

In 2023, Wu *et al.*^253^ introduced a series of bimetallic Ln-MOFs called Tb/Eu-BTC. These Tb/Eu-BTC frameworks demonstrated adjustable dual emission for DPA ratiometric sensing. The efficient energy transfer from Tb^3+^ to Eu^3+^ produced a strong red emission at 616 nm, even with a high proportion of Tb^3+^ in the frameworks. When DPA was introduced, it blocked the energy transfer between Tb^3+^ and Eu^3+^ nodes, resulting in an emission at 544 nm and generating a ratiometric FL response. As a new ratiometric FL probe, Tb/Eu-BTC showed an excellent working range with DPA concentrations ranging from 50 nM to 3 μM, along with a low LOD of 4.9 nM, as shown in Fig. 16.

**Fig. 17:**
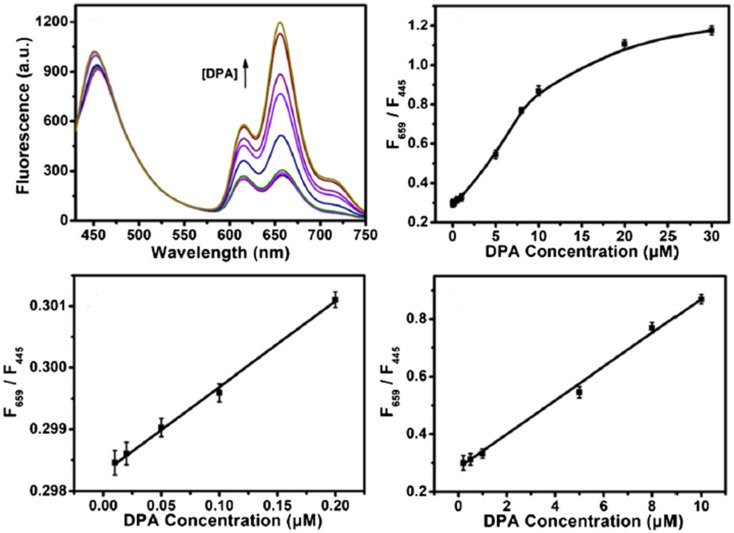
Schematic of the Zn-MOF/CD-based probe for ratiometric FL detection of DPA. Adapted from ref. 254 with permission. Copyright 2021, Springer.

In 2021, Bao *et al.* developed a ratiometric probe for DPA based on Zn-MOF and CDs. The FL intensity at 659 nm increased due to the release of the organic ligand TCPP, which occurred because of the selective interaction between DPA and Zn^2+^ in the MOFs. CDs served as a reference signal at 445 nm, remaining largely unchanged and allowing for self-calibration in DPA sensing. The ratio F_659_ to F_445_ as a function of DPA concentration demonstrated strong linear relationships in the ranges of 0.01–0.2 μM and 0.2–10 μM, with a LOD of 7 nM. This approach was applied to determine DPA in spiked human serum samples, suggesting a novel, simple, and selective strategy for DPA detection,^254^ as shown in Fig. 17.

In this section, we explore in greater detail the most frequently used ratiometric sensing platforms based on luminescent MOFs, as summarized in Table 3.

**Table 3:** List of selected luminescent MOFs, real samples, dual probes, dynamic range, and LOD values

Sample	Dual probes		Dynamic range	LOD	Ref.
	Response	Reference			
Tap water, urine	Blue and green Gd/Tb-MOF	—	0–0.21 mM	1.03 μM	255
Tap and river water	Green Tb^3+^@UIO-67	Blue UIO-67	0.3 to 6 μM	36 nM	256
Lake water	Red and green Tb/Eu-MOF	Blue 2-hydroxyterephthalic acid	0.05 to 20 μM	1.5 nM	257
Tap water and urine	Red and green Tb/Eu-MOF	—	0–800 nM. 20–100 μM	5.9 nM 0.17 μM	258
Spore and water sample	Green Eu-MOF@Tb	Red Eu-MOF@Tb	0.2–10 μM	60 nM	259
Bovine serum	Green Tb-MOFs	Blue Si NPs	0.025 to 3 μM	5.3 nM	122
Human serum	Red and green Tb/Eu@bio-MOF	—	100 to 500 nM	34 nM	260
Human serum	Red and green Eu/Tb-Hddb	—	0–100 μM	0.8494 μM	261

### 6.2 Single probe sensing

The FL detection of LMOFs mainly depends on observing the changes in FL intensity at a specific emission peak of an individual LMOF.^85^ This change happens when the luminescent MOF is exposed to a single excitation wavelength, yielding results for target detection.

In 2023, Yang *et al.* developed a probe utilizing a three-dimensional Cd-based MOF for detecting DPA in bovine serum samples. This probe demonstrates sensitivity with a detection limit of 3.04 μM. Consequently, this study introduces a novel approach for creating transition metal organic framework fluorescent sensors aimed at detecting DPA.^262^

In 2023, Guo *et al.*^263^ developed a novel lanthanide-doped probe (His@ZIF-8/Tb^3+^) for monitoring DPA. This probe demonstrated a satisfactory working range from 0.08 to 10 mM and a LOD of 0.02 mM. It was successfully applied to human urine and bovine serum samples, achieving an excellent recovery range of 98% to 103.2%.

Another study in 2023 involved the preparation of three novel MOFs used as probes for detecting DPA. The LOD for each MOF was determined to be 1.01 × 10^−6^ M (MOF 1), 1.17 × 10^−6^ M (MOF 2), and 2.07 × 10^−6^ M (MOF 3), with a dynamic range for all of them spanning from 0 to 0.991 mM. The probe was applied in fetal bovine serum. Finally, all three types of MOFs can be used as sensors to detect DPA, characterized by high selectivity and sensitivity, and rapid response.^264^

**Fig. 18:**
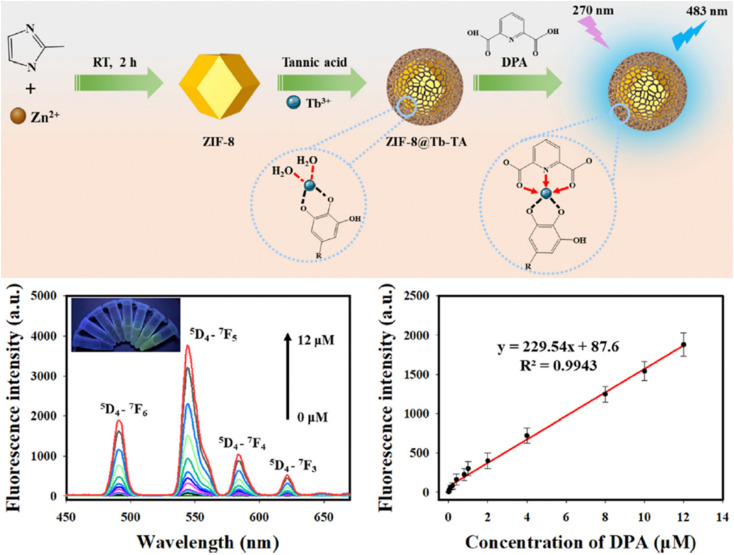
Schematic diagram of the preparation and application of the ZIF-8@Tb–TA probe. Adapted from ref. 265 with permission. Copyright 2023, Royal Society of Chemistry.

Zuo *et al.* prepared a new lanthanide-doped probe by coordinating Tb^3+^ ions with tannic acid (TA)-coated ZIF-8 (ZIF-8@Tb–TA). The probe exhibits a LOD of 12.3 nM, with a working range of 0 mM to 12.0 mM, and was also applied in bovine serum samples.^265^ The overall preparation and application are shown in Fig. 18.

Finally, Deng *et al.*^266^ developed a dual-mode fluorometric/colorimetric sensor for detecting DPA. The FL of Fe-MIL-88NH_2_ was quenched by Cu^2+^, but DPA could restore it due to its strong chelation with Cu^2+^. The FL recovery of Fe-MIL-88NH_2_ and the absorbance change at 652 nm served as analytical signals for dual-mode DPA detection. The fluorometric mode exhibited linear responses within 10–60 μM and 60–160 μM, with a detection limit of 1.46 μM. The colorimetric mode exhibited a linear range of 5–25 μM and a detection limit of 3.00 μM. Overall, this dual-mode approach effectively detected DPA in water samples, indicating its significant potential for disease prevention and environmental monitoring.

### 6.3 Visual detection method

Visual detection has consistently captivated the interest of analytical chemists.^267^ It is typically linked with simple and low-cost instruments, rapid detection, minimal consumption of samples and reagents, and portability for on-site analysis. Currently, high-throughput, rapid discrimination visual tests are crucial in numerous fields.^81,268–270^ Therefore, developing sensitive and selective methods for visually detecting DPA in serum and water samples is extremely important.

**Fig. 19:**
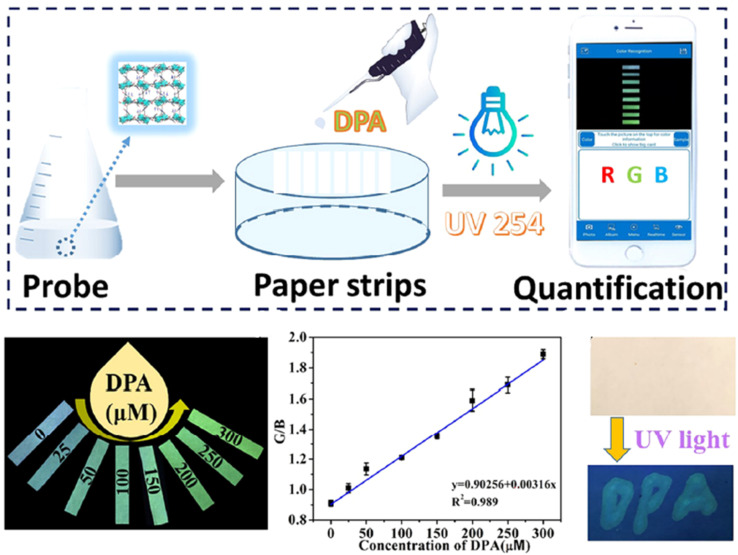
Visual sensing platform using Tb-MOF for DPA detection. Adapted from ref. 271 with permission. Copyright 2023, Elsevier.

Zhang *et al.*^271^ (2023) detected DPA visually using a Tb-MOF probe. The probe shows high selectivity for sensing DPA down to 1.7 μM and exhibits a noticeable luminescence color change that is visible to the naked eye. The ratio of green to blue color (G/B) is directly related to the concentration of DPA. Specifically, the G/B ratio correlates well with DPA concentration in the working range of 0–300 μM, with a LOD of 7.8 μM for visual detection, as shown in Fig. 19. The probe is also effective for detecting DPA in tap water, rainwater, and human serum.

Another study presented a compact visual detection device for DPA based on the Tb-MOF that includes a mini-UV lamp, a smartphone, a paper microchip, and a dark box. This portable visual assay method, utilizing a paper microchip and smartphone-integrated mini-device, achieved a qualification limit of 0.48 μM and was also applied to serum samples.^272^

In addition, Shen *et al.*^273^ prepared novel Eu^3+^/Tb^3+^-MOFs with three ligands for DPA detection, achieving limits of detection of 0.248 μM, 0.874 μM, and 2.277 μM, which were also applied in human serum samples. This study uses a paper-based assay, suggesting that paper-based sensors can be used for rough field detection of DPA, observable through the naked eye. The paper-based MOF sensors can display emission color changes depending on the concentration of DPA, offering an on-site field detection method for DPA.

**Fig. 20:**
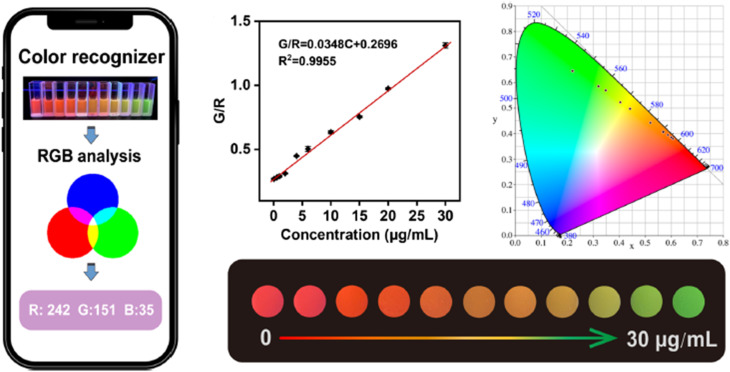
Schematic diagram of the Eu-Tb-MOF for visual detection of DPA. Adapted from ref. 274 with permission. Copyright 2023, Springer.

Wang *et al.*^274^ developed a smartphone-integrated ratiometric fluorescent sensing platform based on a bimetallic MOF (Eu and Tb-MOF) for monitoring the concentration of DPA in the range of 0.06–30 μg mL^−1^, as shown in Fig. 20. The probe was successfully applied to real samples, including human serum samples.

Dashtian *et al.*^93^ (2024) introduced an innovative method where green and yellow emissive N-doped CDs are incorporated into bio-MOFs that are enhanced with functional groups derived from adenine and trimesic acid (BTC) linkers. This results in an exceptional fluorescent sensor capable of detecting DPA within a working range of 0.5 to 75.0 μM. The sensor demonstrates outstanding performance with an extraordinarily low detection limit of 0.16 μM for DPA. Successful practical applications of the sensor have enabled rapid and precise analysis of DPA in spiked urine and water samples. Additionally, this low-cost, quick and user-friendly visual fluorescent sensor facilitates preliminary qualitative analysis of DPA visible to the naked eye.

**Fig. 21:**
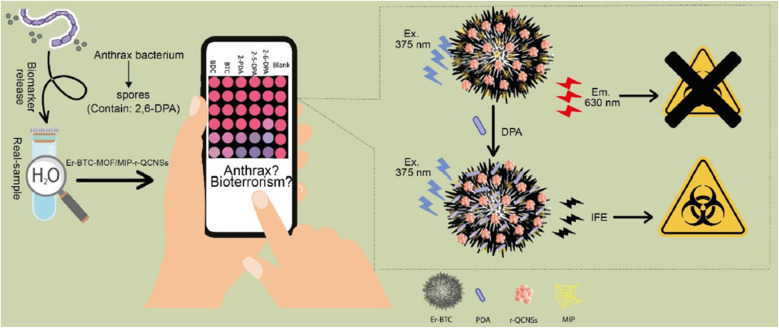
A schematic representation of the probe for DPA detection. Adapted from ref. 275 with permission. Copyright 2023, Royal Society of Chemistry.

In 2023, Norouzi *et al.*^275^ developed a FL sensor probe based on Er-BTC MOF for the visual detection of DPA. They created a paper test strip that combines online UV excitation with a smartphone, forming a DPA signal-off sensing platform. This fluorometric visual paper-based biosensor offers a wide linear range for DPA detection (10–125 μM), with LOQ and LOD values of 4.32 and 1.28 μM, respectively. As a proof of concept, the sensor was effectively used to monitor DPA in real samples of tap water and urine, as depicted in Fig. 21.

In 2024, Wang *et al.* developed a ratiometric FL sensor based on LnMOFs for the sensitive and selective recognition of DPA. The thoughtfully engineered Eu-MOF demonstrated exceptional sensitivity, robust stability, and remarkable resistance to interference for detecting DPA. It also showed a clear color transition from red to blue with increasing DPA levels under UV light. Notably, the Eu-MOF probes also performed well in detecting DPA in fetal calf serum and tap water.^276^

**Fig. 22:**
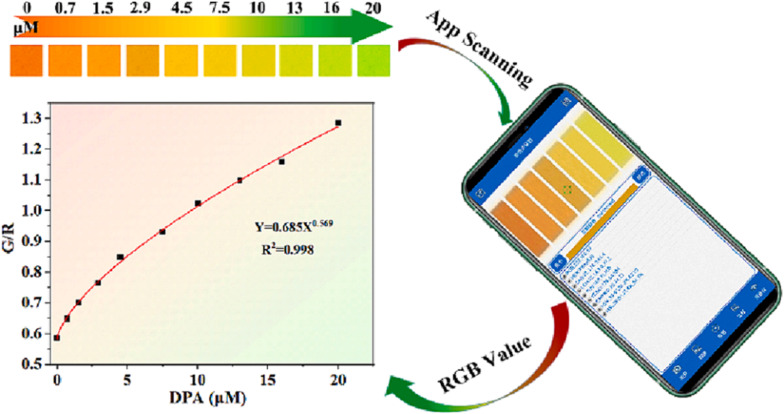
Visual assay of DPA. Adapted from ref. 277 with permission. Copyright 2024, Elsevier.

Gou *et al.*^277^ developed a multi-color fluorescent probe using halloysite nanotubes combined with fluorescent dyes and porous MOFs for highly sensitive DPA detection, with a limit of detection of 11.27 nM. This probe allowed for rapid, accurate, and selective DPA detection. They also created a portable, cost-effective visual sensor by immobilizing the probe on filter paper, which, when paired with a smartphone, enabled real-time, intuitive DPA detection with a minimum concentration of about 0.70 μM, as shown in Fig. 22.

**Fig. 23:**
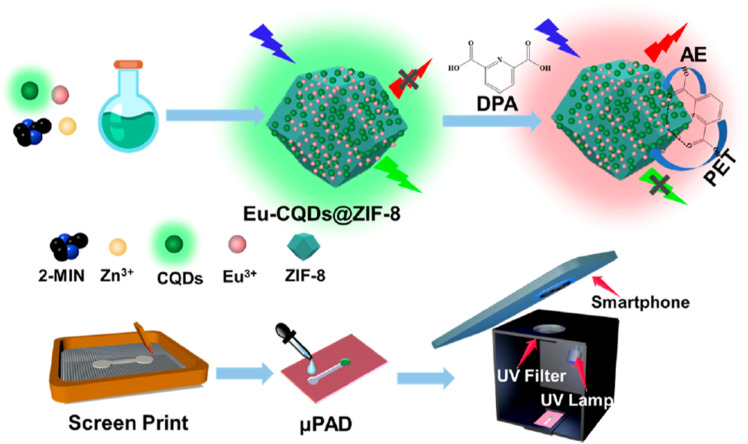
Schematic depiction of the preparation process for (A) Eu-CQDs@ZIF-8 and (B) μPAD used in DPA detection. Adapted from ref. 278 with permission. Copyright 2024, American Chemical Society.

In 2024, Wang *et al.*^278^ introduced an innovative paper-based ratiometric fluorescence sensor platform utilizing Eu^3+^-doped carbon quantum dots (CQDs) embedded within ZIF-8 (Eu^3+^-CQDs@ZIF-8), which was applied in human serum samples. This platform enables rapid detection of DPA. Upon exposure to DPA, the sensor platform exhibited a noticeable color change from green to red in the μPAD detection zones. This color transition corresponds to increasing concentrations of DPA, facilitating quantitative analysis by converting the color signals into R/G ratios. The platform demonstrates a strong linear response across varying DPA concentrations (1–60 μM), achieving sensitivity levels comparable to those of established fluorescence spectroscopy techniques. This underscores the platform's effectiveness in meeting practical sample detection needs, as illustrated in Fig. 23.

## 7. Concluding remarks and future direction

This review provides a comprehensive overview of the methodologies for developing MOF-based luminescent sensors, emphasizing the advantages of MOFs in sensor design. These advantages include their diverse emissive properties, which may arise from metal ions, organic ligands, or guest species, as well as their structural diversity, which can be finely tuned through modifications of ligands, metal ions, or reaction conditions. Additionally, their morphology can be adapted through adjustments to reaction parameters. Recently, there has been growing interest in the application of luminescent MOF-based materials as sensors, owing to their exceptional features, such as high porosity, large surface area, and controlled structure, all of which enhance their suitability for sensing applications.

Researchers are employing various synthetic approaches to create luminescent MOFs, yet significant challenges remain, particularly concerning the practical use of these materials for detecting DPA in complex matrices. Therefore, future research should prioritize the development of robust, reusable, and highly sensitive luminescent MOF probes specifically designed for DPA detection. This review aims to summarize the current state of luminescent MOF-based sensors for DPA detection while highlighting the need for advancements in material synthesis techniques and modification strategies to overcome existing challenges. Furthermore, understanding the influence of these materials on experimental outcomes and their applicability to real-world samples is crucial.

In conclusion, luminescent MOF-based sensors have shown significant potential for DPA detection, but ongoing research is necessary to develop more innovative and reliable sensors to enhance detection efficiency. It is hoped that this review will inspire further interest in the development and refinement of luminescent MOF-based sensors, leading to their broader application in DPA detection in the near future.

## Data availability

Data sharing is not applicable to this article as no datasets were generated or analysed during the current study.

## Conflicts of interest

There are no conflicts to declare.
